# Enhanced mortality prediction in pediatric sepsis using NGAL: A comparison with PRISM III scores in critical care settings

**DOI:** 10.1007/s00431-025-06017-8

**Published:** 2025-02-15

**Authors:** Marwa Ibrahem AbdelRazic, Gehan Lotfy Abdel Hakeem, Mina Sobhy Hanna, Omima M. Mohamed, Ibtehal Saad Abuelela

**Affiliations:** 1 Pediatrics Department, Faculty of Medicine, Minia University, Minia, Egypt; 2 Clinical Pathology Department, Faculty of Medicine, Minia University, Minia, Egypt

**Keywords:** Pediatric patients, Prognostic value and severity, Sepsis, Serum neutrophil gelatinase-associated lipocalin

## Abstract

Sepsis is a critical condition that disrupts metabolic, physiological, and immune functions, often resulting in severe complications such as multi-organ failure and increased mortality. Neutrophil gelatinase-associated lipocalin (NGAL) has emerged as a promising biomarker for infection and inflammation, offering potential advantages for early mortality prediction. This study compared the predictive value of serum NGAL levels with pediatric risk of mortality III (PRISM III) scores in critically ill pediatric patients with sepsis. A prospective cohort study was conducted at a tertiary hospital from September 2022 to March 2023, involving 75 pediatric patients diagnosed with sepsis, septic shock, or multi-organ dysfunction syndrome (MODS), along with 25 healthy controls. Serum NGAL levels were measured within the first hour of PICU admission and analyzed alongside PRISM III scores to evaluate their correlation with mortality and sepsis severity. The results demonstrated that serum NGAL levels were significantly elevated in septic patients compared to controls, with the highest levels observed in those with MODS. NGAL showed greater sensitivity and specificity for predicting mortality than PRISM III scores, with ROC curve analysis revealing that NGAL levels > 599 mg/ml were strongly associated with increased mortality risk (sensitivity 70.4% and specificity 50%). Multivariate analysis confirmed NGAL as an independent predictor of mortality, outperforming PRISM III scores in identifying severe cases.

*Conclusion*: Serum NGAL is a valuable biomarker for early prediction of mortality and sepsis severity in pediatric patients, providing faster and more accurate assessments than PRISM III scores. Its integration into clinical practice may enhance decision-making in pediatric critical care settings, allowing for timely interventions and improved patient outcomes.

**Table:** 

**What is Known:** • *Pediatric risk of mortality III (Prism III) scores is widely used to predict sepsis severity and mortality in pediatric intensive care units, but requiring 12-24 hours to complete. Neutrophil is an established biomarker for inflammation and infection with a potentially anti-pathological value in the neutrophil gelatinus-lipocalin (NGAL) sepsis.*
**What is New:** • *Serum NGAL levels, PICU is measured within the first hour of entry, prism III score in pediatric patients in predicting mortality and severity of sepsis. > An NGAL cutoff of 599 mg/mL is significantly associated with mortality risk, which provides a rapid, independent and more immediate immunity tool for important care decision making.*

**Supplementary Information:**

The online version contains supplementary material available at 10.1007/s00431-025-06017-8.

## Introduction

Sepsis is a life-threatening condition that is defined by a dysregulated host response to infection, leading to severe physiological, metabolic, and immune disturbances. Severe sepsis progresses to multi-organ dysfunction syndrome (MODS) when organ failure occurs, while septic shock is defined by acute circulatory collapse and persistently low arterial pressure despite adequate volume resuscitation [1, 2]. Children’s bloodstream infections are a major global health problem. It accounts for 10–20% of child deaths. With a proportion of deaths occurring in the early period after diagnosis [3–5], these alarming statistics highlight the urgent need for precise, early intervention to improve outcomes and reduce mortality [6].

The pediatric risk of mortality score III (PRISM III) is a highly recognized tool. It is used to evaluate illness severity and forecast mortality in the pediatric intensive care unit (PICU) [7]. Developed by Pollack et al., laboratory results and neurological conditions [8]; however, the PRISM III score requires 12–24 h to complete. This limits its utility for rapid decision-making in time-sensitive clinical situations [9] and biomarkers. That helps detect the severity of infection and the risk of death early. Identification remains an important research priority [10].

Neutrophil gelatinase-associated lipocalin (NGAL) has emerged as a prospective biomarker for forecasting the severity and mortality of sepsis. NGAL, a 25-kDa protein, was first discovered in neutrophils for its antibacterial capabilities. Epithelial cells also generate it in reaction to oxidative stress and inflammation [11, 12]. NGAL is strongly associated with important proinflammatory cytokines, including IL-6, IL-10, and TNF-12. NGAL expression significantly increased during inflammatory transformation, as observed in studies by Otto et al. [13] and Kim et al. [14]. Higher levels of NGAL were shown in septic patients. This is especially true in people with severe disease [13, 14]. Furthermore, plasma NGAL is associated with the severity of inflammation. It is therefore a valuable tool for clinical assessment.

This research sought to assess the significance of blood NGAL levels as an early biomarker for infection severity and mortality risk in pediatric patients upon admission. This study focuses on treatment in the PICU and aims to improve outcomes for critically ill children through timely support and effective clinical intervention.

## Patients and methods

### Study design

The research was executed as a prospective cohort study at a tertiary care hospital from September 2022 to March 2023. Participants were enrolled upon admission to the PICU with serum NGAL levels measured as a test. Primary exposure was within the first hour of admission and then further follow-up over time to assess outcomes, including mortality and severity of sepsis. Healthy controls were included to maintain baseline NGAL levels for comparison.

### Study population

The research had 100 individuals, consisting of 75 pediatric sepsis patients hospitalized to the pediatric intensive care unit (PICU) and 25 healthy controls. Patients were categorized. Infection severity is categorized into three classes.

- *Sepsis group*: 25 patients.
- *Severe sepsis group*: 25 patients.
- *Multi-organ dysfunction syndrome (MODS) group*: 25 patients.

The control group included 25 healthy children matched for age and sex.

### Inclusion and exclusion criteria

1. Inclusion criteria:
  Children below 12 years old admitted to the PICU fulfill the ACCP/SCCM criteria for sepsis [15].
2. Exclusion criteria:
  Children over 12 years of age.
  Delay in blood sampling beyond the first hour of PICU admission.
  Lack of parental consent.

### Data collection

The following information was gathered for examination.

1. Medical history: Detailed documentation of age, gender, residence, and duration of illness. and symptoms shown
2. Physical examination: comprehensive assessment of the cardiovascular system. Respiratory system, nervous system, and other systems

### Diagnostic criteria for septicemia

1. *Temperature*: Axillary temperature > 38.5 °C or < 36 °C.
2. *Heart and respiratory rates*: Elevated rates based on age norms.
3. *Additional indicators*:
  o Positive blood culture.
  o Abnormal leukocytes count for age.
  o Immature neutrophils ≥ 10% [16].

### Diagnostic criteria for septic shock

The presence of any of the following signs indicating poor perfusion despite adequate fluid resuscitation [16]:

- Altered mental status.
- Prolonged capillary refill time.
- Weak or diminished pulses.
- Cold, mottled extremities.
- Urine output < 1 ml/kg/hour.
- Persistent hypotension.

## Methodology

### Study procedures

#### Blood sampling protocol

Standard laboratory tests included blood cultures, prothrombin time (PT), prothrombin concentration (PC), C-reactive protein (CRP), and complete blood count (CBC).

- *Blood cultures*: Venous blood samples were collected aseptically using the BACTEC system (Becton Dickinson Diagnostic Instrument Systems, Sparks, Maryland, USA). Subcultures were performed for positive cases to identify pathogens and perform antimicrobial susceptibility testing (AST) using the VITEK-2 system (bioMérieux, USA).
- *PT and PC*: Turbo-densitometric analysis was conducted using the LABiTec CoaDATA 4004 analyzer and PT-Reagent kit (Biochemical Technology GmbH, Germany) according to manufacturer instructions.
- *CBC*: CBC was analyzed using a five-part differential Celltac G automated hematology analyzer (Nihon Kohden Corporation).
- *CRP*: CRP was measured using a kinetic assay with reagents from GENRUI Biotech Inc. (China), following manufacturer guidelines.

#### PRISM III score

Data for the PRISM III scoring system were obtained from clinical and laboratory records, including parameters such as blood pressure, glucose level, platelet count, white blood cell count, pulse oximetry, neurological status, and pupil light reflex [8]. Scores were calculated using an online PRISM III calculator (www.medal.org) [17].

Interpretation of scores and corresponding mortality risks were as follows:

- 0–5: 11%
- 6–10: 23%
- 11–15: 40%
- 16–20: 61%
- 21–25: 78%
- 26–30: 89% [8].

#### Assessment of NGAL serum levels

Serum NGAL levels were assessed during the first hour of PICU admission for all patients and controls via a dual monoclonal antibody sandwich ELISA (human neutrophil gelatinase-associated lipocalin ELISA reagent; Glory Bioscience, Del Rio, TX) [18].

#### Outcomes measured

- *Primary outcome*: Mortality prediction using serum NGAL levels and PRISM III scores.
- *Secondary outcome*: Correlation of NGAL levels with sepsis severity (sepsis, severe sepsis, and multi-organ failure).

### Ethical considerations

The Institutional Review Board (IRB) of the Faculty of Medicine at Minia University, Minia, Egypt, granted approval for the research on September 19, 2022, under Approval No. 388:2022. All study techniques adhered to the principles established in the Declaration of Helsinki. Informed written permission was acquired from the parents or legal guardians of all involved minors.

### Data analysis

This study’s statistical analysis used SPSS version 26 to assess the predictive significance of blood NGAL levels in relation to PRISM III scores for predicting death and sepsis severity in critically sick pediatric patients. Descriptive statistics were used to summarize parametric and non-parametric data. Parametric variables were reported as *mean* ± standard deviation (*SD*) and range, while non-parametric variables were characterized by median and interquartile range (*IQR*). This offers a thorough insight into the primary patterns and distribution of the gathered data.

Inferential statistical techniques were used to analyze correlations and disparities among groups. The chi-square test was used to examine categorical variables. It elucidates the correlation among groups, infection severity, and clinical consequences. The choice of statistical tests for continuous variables is dictated by the data’s distribution. Parametric data were examined using analysis of variance (ANOVA) for comparative study across several groups. A Bonferroni test was then used to ascertain significant pairwise differences. The Kruskal–Wallis test was conducted on non-parametric data. It was used to evaluate disparities among groups. The two groups used the Mann–Whitney *U* test for comparison.

The predictive accuracy of serum NGAL levels and PRISM III ratings for mortality and multiple organ failure was evaluated by receiver operating characteristic (ROC) curve analysis. This examination — the diagnostic performance of the biomarkers was assessed using area under the curve (*AUC*), sensitivity, specificity, and positive predictive value (*PPV*). By delineating the necessary indicators, *AUC* values over 0.7, including negative predictive value (*NPV*), were deemed to signify adequate predictive ability. It underscores the therapeutic significance of NGAL as a rapid diagnostic instrument.

A multivariate logistic regression analysis was conducted to discover further independent determinants of mortality. This investigation included serum NGAL levels, PRISM III scores, and other laboratory indicators. To assess the cumulative impact on mortality risk is to guarantee the integrity of the finding. The criterion for statistical significance was established at *p* < 0.05 for all analyses.

Graphs and visualizations were generated using SPSS to illustrate significant correlations between variables, including the correlation between NGAL levels and infection severity and mortality outcomes. These visual instruments enhance clarity and facilitate data comprehension. The research thoroughly assessed the prediction capacity of blood NGAL concentrations and the PRISM III score. Employing a systematic and comprehensive analytical methodology, it underscores its efficacy in enhancing clinical decision-making within pediatric critical care environments.

## Results

A total of 100 people participated in the study. This included 75 pediatric sepsis patients and 25 healthy controls. The patient group was further subdivided into three groups based on the severity of their infection: 25 with sepsis, 25 with severe sepsis, and 25 groups with multiple organ dysfunction syndrome (MODS) did not find a significant difference 0.75) (Table 1).

**Table 1:** Baseline characteristics, laboratory data, and mortality rates among study groups with post hoc statistical comparison

Variable	Sepsis (*n* = 25)	Severe sepsis (*n* = 25)	MODS (*n* = 25)	Control (*n* = 25)	*p*-value	Post hoc analysis
Age (years)	6.9 ± 3.0	6.7 ± 2.6	6.8 ± 4.2	6.5 ± 3.2	0.98	-
Gender						
Males (%)	40% (10)	48% (12)	56% (14)	48% (12)	0.75	-
Females (%)	60% (15)	52% (13)	44% (11)	52% (13)		
s.NGAL (mg/ml)	532.56 ± 26.59	671.8 ± 49.71	1534.12 ± 467.47	365.4 ± 60.50	< 0.001*	P1 = 0.16; P2 < 0.001; P3 = 0.05; P4 < 0.001; P5 < 0.001; P6 < 0.001
Hb (g/dl)	9.82 ± 0.52	8.56 ± 0.55	8.4 ± 0.94	11.55 ± 0.91	< 0.001*	P1 < 0.001; P2 < 0.001; P3 < 0.001; P4 = 0.89; P5 < 0.001; P6 < 0.001
TLC (× 10⁹/L)	12.71 ± 1.31	14.8 ± 1.25	23.97 ± 6.67	8.02 ± 1.16	< 0.001*	P1 = 0.15; P2 < 0.001; P3 < 0.001; P4 < 0.001; P5 < 0.001; P6 < 0.001
PLT (× 10⁹/L)	158.56 ± 7.53	138.36 ± 8.76	91.48 ± 35.49	311.2 ± 51.97	< 0.001*	P1 = 0.12; P2 < 0.001; P3 < 0.001; P4 < 0.001; P5 < 0.001; P6 < 0.001
RBS (mg/dl)	171.56 ± 17.76	268.52 ± 20.47	279.04 ± 173.84	-	P1 = 0.003*	
PRISM III score	16.04 ± 3.43	24.96 ± 2.81	30.32 ± 4.09	-	< 0.001*	P1 < 0.001; P2 < 0.001; P3 < 0.001; P4 < 0.001; P5 < 0.001; P6 < 0.001
CRP (mg/dl)	12 (6–18)	24 (12–48)	48 (48–96)	-	< 0.001*	P1 = 0.01; P2 < 0.001; P3 < 0.001; P4 < 0.001; P5 < 0.001; P6 < 0.001
Mortality						
Survived (%)	40% (10)	28% (7)	16% (4)	-	0.18	-
Died (%)	60% (15)	72% (18)	84% (21)	-		

*Asterisks *(*) in Table 1 indicate statistical significance in post hoc analysis comparisons, with *p*-values < 0.05 denoting significant differences between the compared groups

Post hoc analysis explanation: P1: *p*-value between sepsis and severe sepsis. P2: *p*-value between sepsis and multi-organ failure. P3: *p*-value between sepsis and control. P4: *p*-value between severe sepsis and multi-organ failure. P5: *p*-value between severe sepsis and control. P6: *p*-value between control and multi-organ failure

Serum NGAL levels were significantly elevated in septic patients compared to healthy controls, with the highest levels observed in the MODS group (1534.12 ± 467.47 mg/ml) compared to the severe sepsis group (671.8 ± 49.71 mg/ml) and the sepsis group (532.56 ± 26.59 mg/ml) (*p* < 0.001) (Table 2). Post hoc analysis confirmed significant differences between these groups, with NGAL levels increasing proportionally with sepsis severity. Furthermore, laboratory findings revealed notable variations in other markers, including CRP, total leukocyte count (TLC), platelet count, and random blood sugar (RBS), which were all significantly altered in MODS patients compared to other groups (*p* < 0.001 for all) (Table 1).

**Table 2:** Comparison of s. NGAL, PRISM III scores, and other lab data regarding patients’ mortality

Variable	Survived *N* = 21	Died *N* = 54	*p* value
S. NGAL (mg/ml)	642.52 ± 148.1	1017.94 ± 574.1	< 0.001*
Prism III score	21.33 ± 6.9	24.72 ± 6.65	**0.05**
CRP	12 (9–24)	24 (21–48)	0.003*
HB	8.8 ± 1.03	8.9 ± 0.90	**0.79**
TLC	16.3 ± 7.2	17.4 ± 5.9	**0.48**
Platelet	146 ± 17.3	123 ± 38.5	0.001*
RBS	216.9 ± 66.8	248.5 ± 123.9	**0.16**

Analyzed by independent sample *t*-test for parametric data and Mann–Whitney test for non-parametric data; numerical parametric data expressed as *mean* ± *SD* and analyzed by independent sample *t*-test and numerical non-parametric data expressed as median (*IQR*) and analyzed by Mann–Whitney test.

Bolded values in Table 2 represent statistically significant differences (*p* < 0.05) based on independent sample *t*-tests for parametric data and Mann-Whitney U tests for non-parametric data

*Significant difference at *P* value < 0.05 s.

*NGAL* serum neutrophils gelatinase-associated lipocalin, and PRISM III: pediatric risk of mortality III score

PRISM III scores also demonstrated significant differences across the sepsis severity groups, with the highest mean score in the MODS group (30.32 ± 4.09), followed by the severe sepsis group (24.96 ± 2.81) and the sepsis group (16.04 ± 3.43) (*p* < 0.001) (Table 1). However, when comparing survivors and non-survivors, PRISM III scores were less effective in distinguishing outcomes (*p* = 0.05) (Table 2).

**Fig. 1:**
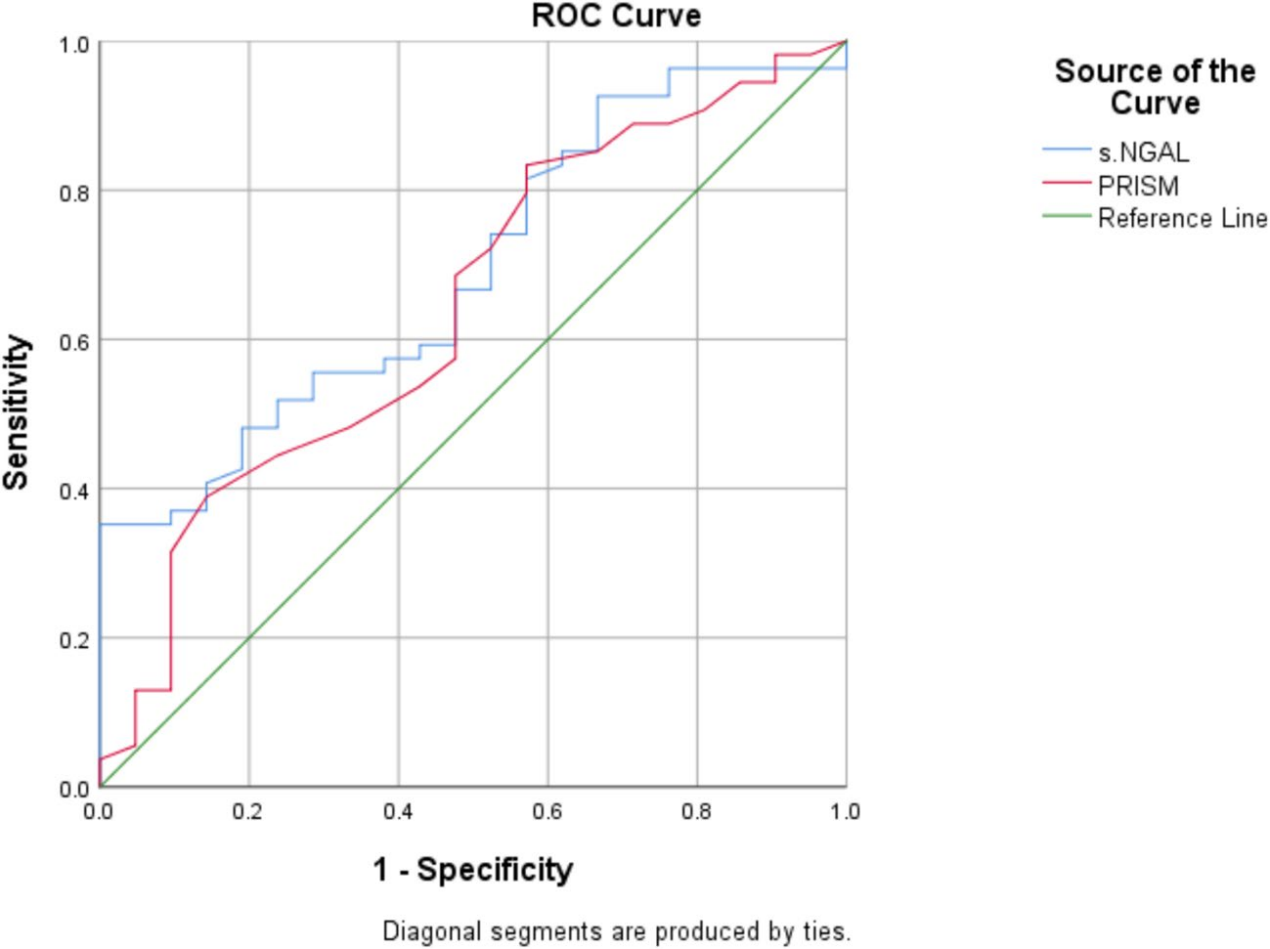
ROC curve analysis for NGAL and PRISM for prediction of mortality

Mortality analysis revealed that NGAL levels were significantly higher in non-survivors (1017.94 ± 574.1 mg/ml) compared to survivors (642.52 ± 148.1 mg/ml) (*p* < 0.001) (Table 2). ROC curve analysis showed that NGAL outperformed PRISM III scores in predicting mortality, with an *AUC* of 0.70 for NGAL compared to 0.64 for PRISM III scores (*p* = 0.01 vs. *p* = 0.05). An NGAL cut-off value of > 599 mg/ml demonstrated a sensitivity of 70.4% and a specificity of 50% for mortality prediction, with corresponding positive and negative predictive values of 77.6% and 45%, respectively (Fig. 1).

Multivariate binary logistic regression analysis confirmed NGAL as an independent predictor of mortality (odds ratio [*OR*]: 1.01; 95% confidence interval [*CI*] 1.001–1.04; *p* = 0.01), whereas PRISM III scores and other laboratory markers did not retain statistical significance in the adjusted model (Table 3).

**Table 3:** Multivariate regression analysis for predictors of mortality among studied cases

Variable	Univariate analysis			Multivariate analysis		
	*OR*	95% *CI*	*p* value	*OR*	95% *CI*	*p* value
**S. NGAL (mg/ml)**	0.99	0.997–1	0.02*	1.01	1.001–1.04	0.01*
**Prism III score**	0.93	0.858–1.003	0.05	0.58	0.18–1.8	0.37
**CRP**	0.97	0.94–0.99	0.01*	0.96	0.87–1.6	0.44
**Platelet**	1.03	1.005–1.059	0.01*	0.59	0.23–1.4	0.59

*OR* odds ratio, *CI* confidence interval, *s. NGAL* serum neutrophil gelatinase-associated lipocalin, *PRISM III* pediatric risk of mortality III score

* Significant level at *p* value < 0.05

The predominant etiologies of sepsis in patients were pneumonia (60%), gastroenteritis (28%), and central nervous system infections (12%). Bacterial cultures revealed *Klebsiella pneumoniae*, *Escherichia coli*, and *Staphylococcus aureus* as the primary pathogens in patients with severe sepsis and MODS, whereas 28% of cases had no bacterial growth.

## Discussion

From September 2022 to March 2023, we did a prospective cohort study. For the control group, we chose 25 kids who seemed healthy and 75 kids with sepsis from the pediatric critical care unit.

Our study looked at children with sepsis who were admitted to the pediatric critical care unit at our hospital, which sought to establish if serum NGAL was associated with sepsis severity and mortality risk. Comparing this link to the PRISM III score, which has been shown in several studies to accurately predict the death rate of admitted patients with high specificity and sensitivity [19, 20], was also done.

In our study, Young patients in intensive care who had severe sepsis and septic shock had much higher amounts of Sera NGAL than healthy controls. In addition, children with severe sepsis had significantly lower blood NGAL levels than severely ill children with septic shock, indicating that the serum level increased in direct association with the severity of sickness in those children. We found that there was a statistically significant difference in blood NGAL levels between the survivors and the people who did not survive. This meant that people with higher amounts of NGAL were also more likely to die.

In line with what was found in Kassam et al. [21], we found that serum NGAL levels were greater in the group of children with multiple organ failure compared to the other groups of children with sepsis.

When comparing severely ill children who died to those who survived, reference [22] found that the levels of NGAL were significantly elevated during admission for the latter group. A study done by Kümpers et al. also found the median and interquartile range of NGAL levels to be higher in the nonsurvivor group compared to the survivors [23]. Furthermore, it was asserted by Ridder et al. [24] that plasma and urine levels of NGAL are substantially elevated in patients who do not survive when confined to the intensive care unit in comparison to children who do survive.

When comparing the mean PRISM III scores of different sepsis groups, we discovered that multiorgan failure had the highest score with a significant difference between the three sepsis severity groups, but when comparing the scores of survivors and non-survivors, we did not find a significant difference.

We performed multivariate regression analysis which confirmed that the 1st hour admission serum NGAL was the only predictor of mortality among other laboratory markers and PRISM III score.

To determine the prognostic power of serum NGAL in predicting mortality, ROC curve analysis was performed, which concluded that only serum NGAL with a cut-off point > 599 mg/ml had a significant role in predicting mortality with a sensitivity of 70.4% and specificity of 50% in predicting mortality.

These results come in accordance with research done in a tertiary hospital in Saudi Arabia [25], and they showed that PRISM III score mortality predictions were low regarding, as most of the admitted cases were with pneumonia which is in accordance with our results.

The superiority of NGAL over PRISMIII score regarding sepsis mortality may be related to that most cases were admitted with community-acquired pneumonia, to which NGAL has more sensitivity than other causes of sepsis as ascertained by two studies, which illustrated a robust correlation between the severity of community-acquired pneumonia and NGAL. Kim et al. [26] and Yeh et al. [27] found a strong association between clinical pneumonia severity rating systems and plasma NGAL levels. According to Kim et al. [26], higher NGAL levels were also found to be a significant predictor of death in these populations.

According to our analysis, *Klebsiella* growth was detected in more than half of the samples (52.6%) from the group that experienced multi-organ failure and approximately 47% from the group that experienced severe sepsis. Our findings are consistent with those of Liu et al. [28], who uncovered that *Klebsiella pneumoniae* is a common bacterium and that sepsis caused by this bacterium significantly increases the risk of systemic multi-organ failure. Additionally, we found that approximately 25% of positive culture results are attributable to the growth of *E. coli*, while 15.6% are attributable to the growth of *Staphylococcus aureus*.

Neutrophils, epithelial cells, and liver cells are the targets of inflammatory cytokines. This stimulates the production of NGAL. According to research by Luchtefeld et al. [29] and Cowland et al. [30], to reduce bacterial growth and replication, NGAL secretes siderophores which are produced by bacteria. This is because iron absorption is a general factor in the reproductive ability of pathogens [31].

RSV, *M. tuberculosis*, and *Klebsiella pneumoniae* are the pathogens that NGAL protects the respiratory system from [32–34]. According to Fröderer et al. [35], NGAL mostly acts as a bacteriostat by trapping harmful bacterial siderophores, but it also helps stimulate and differentiate T-cells into Th1s.

## Limitations

The study’s small sample size and single-center design limit generalizability to a broad population and clinical setting. Long-term data to track changes in NGAL levels is lacking and does not take into account confounding factors such as comorbidities and previous treatment. This may affect the results. These findings are specific to children under 12 years of age, limiting their applicability to other age groups. Focusing on serum NGAL alone, evaluation of other biomarkers was not included. The moderate prediction accuracy emphasizes the need for further investigation and combination with additional diagnostic tools.

## Interpretation

Serum NGAL has emerged as a valuable biomarker for predicting infection severity and mortality. It outperforms PRISM III scores in terms of timeliness and accuracy. It can be measured within the first hour of PICU admission. Its levels are related to the severity of the infection, especially multiple organ failure. *Klebsiella pneumonia* resembles bacteria in cases of infection, although the sensitivity and specificity are moderate. However, it increases the potential of NGAL when combined with other clinical and laboratory markers. Make it a practice to stratify risk early on in seriously ill pediatric patients.

## Generalizability

Findings from a single tertiary care hospital may not generalize health care systems with different resources and demographics. This study focused on children under 12. It also limits applicability to the elderly population. Variation in NGAL levels due to genetic, nutritional, or regional factors has not been resolved. And the specific results of the pathogen in particular, *Klebsiella pneumoniae* and *E. coli* have not been resolved, despite these limitations not applying to areas with different bacterial profiles. The ease of measuring NGAL makes it a promising biomarker for resource-limited settings.

## Conclusion

Our research concluded that serum NGAL levels have a statistically significant relationship with sepsis severity and mortality risk. Higher serum NGAL levels at admission are directly associated with increased sepsis severity and mortality in critically ill children in the PICU. Serum NGAL outperformed the PRISM III score as a predictor of outcomes in bacterial sepsis. Unlike the PRISM III score, which requires 12–24 h for calculation, serum NGAL provides immediate prognostic information through a simple blood test, enhancing its utility for urgent clinical decision-making.

## Supplementary Information

Below is the link to the electronic supplementary material.

Supplementary file1 (DOCX 14 KB)

## Data Availability

No datasets were generated or analysed during the current study.

## Declarations

### Authorization for publication.

Parents/legal guardians provided written informed consent.
