# Supplementary material for: Enhanced mortality prediction in pediatric sepsis using NGAL: A comparison with PRISM III scores in critical care settings
Source: Eur J Pediatr. 2025 Feb 15;184(3):201. doi: 10.1007/s00431-025-06017-8 (PMC11829929; doi:10.1007/s00431-025-06017-8)
Supplement: Supplementary file 1 — Supplementary file1 (DOCX 14 KB) [file 431_2025_6017_MOESM1_ESM.docx]

**Table 1s: Comparison of NGAL and PRISM III ROC Analysis**

| **Variable** | **NGAL** | **PRISM III Score** |
| --- | --- | --- |
| **AUC** | 0.70 | 0.64 |
| **Cut-off Value** | >599 mg/ml | >21.5 |
| **Sensitivity** | 70.4% | 68% |
| **Specificity** | 50% | 51% |
| **PPV (Positive Predictive Value)** | 77.6% | 78% |
| **P value** | 0.01* | 0.05* |
| **P-value for AUC Comparison (DeLong Test)** | 0.176 | |

AUC: Area Under Curve CI: Confidence Interval PPV: Positive Predictive Value NPV: Negative Predictive Value *: Significant Level at P value < 0.05

s.NGAL: serum neutrophils gelatinase-associated lipocalin, and PRISM III: pediatric risk of mortality III score.
